# Study on Slipform Paving of Concrete Containing Alkali-Free Accelerators on Roadway Floor

**DOI:** 10.3390/ma17246298

**Published:** 2024-12-23

**Authors:** Yongjing Deng, Guanguo Ma, Zhenjiao Sun, Kang Gao, Hui Ma, Tingting Song, Wenfeng Jia

**Affiliations:** 1 College of Mining and Safety Engineering, Shandong University of Science and Technology, Qingdao 266590, China; dyj473902436@163.com (Y.D.); sunzhenjiao@foxmail.com (Z.S.); skdgk@sdust.edu.cn (K.G.); song17111998@163.com (T.S.); wf980918@163.com (W.J.); 2 School of Safety and Engineering, China University of Mining and Technology, Xuzhou 221116, China; hughie_ma@163.com

**Keywords:** accelerator, concrete performance, orthogonal experiment, width estimation formula

## Abstract

Aiming at the problems of collapse, deformation, and displacement in the concrete paving of roadway floors, this paper adopts the way of adding alkali-free accelerators to the concrete on both sides, through mechanical analysis, single factor experiment, orthogonal experiment, and polynomial fitting method, and determines the relevant parameters of concrete and accelerators in the sliding form paving of roadway floor from two aspects of paving material and size. The results show that the FSA-AF alkali-free liquid accelerator is more suitable for roadway floor paving than the J85 powder accelerator. When the FSA-AF accelerator dosage reaches 8%, the decreasing trend of initial setting time curve tends to be flat. The deformation resistance of concrete containing accelerator is positively correlated with the dosage of the accelerator. Concrete side pressure is positively correlated with pavement paving height. The FSA-AF accelerator can reduce the compressive strength of concrete; the compressive strength and retention rate of concrete at all ages are the highest when the dosage of FSA-AF is 7%. A water–cement ratio of 0.4 and a 9% dosage of accelerator are the optimal combination to meet the four evaluation indexes. According to the width estimation formula, the width of the side concrete should be set to 14 cm.

## 1. Introduction

In the process of paving roadway floor concrete by sliding form pavers, the concrete on both sides is prone to collapse, deformation, and displacement, which seriously affects the paving quality and speed [1,2]. At present, the main methods to solve the problems of concrete collapse and deformation are to adjust the slump of concrete, alter the gradation and ratio of aggregate, or add concrete admixtures to make concrete have a lower slump and better workability [3,4,5,6]. Although adjusting the gradation and concrete admixtures can result in lower slump, it does not improve the initial setting time and rapid solidification performance of concrete. This method only reduces the degree of collapse and deformation of the side concrete and does not fundamentally solve the problem of paving. Moreover, the use of admixtures can affect strength and increase costs. The accelerator is often used in mine concrete spraying, which can significantly improve the initial setting time of concrete and accelerate the hardening process of cement [7]. After the concrete sliding mode paving, the accelerator can accelerate the hydration process, making the cement slurry quickly reach the initial setting state and making the aggregate particles in the concrete solidify and support each other, with a certain early strength. Therefore, concrete can maintain its original shape even after losing the support of the formwork, avoid the problems of edge collapse and deformation in paving, and meet the requirements of low collapse and no deformation of concrete in concrete paving [8,9].

**Figure 1:**
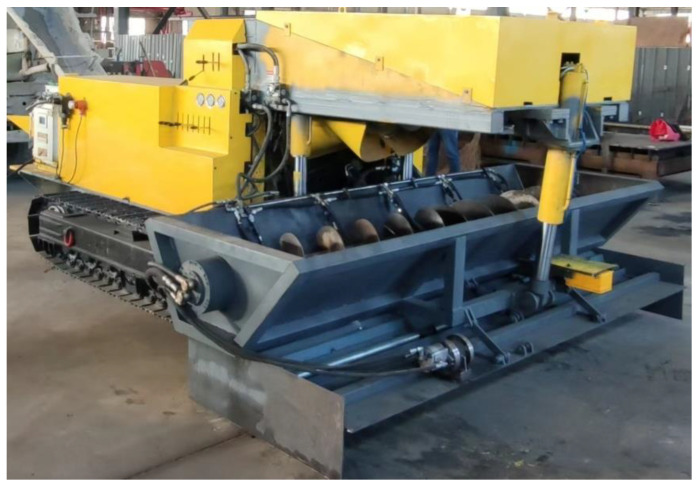
Slip-form pavers for mining concrete.

**Figure 2:**
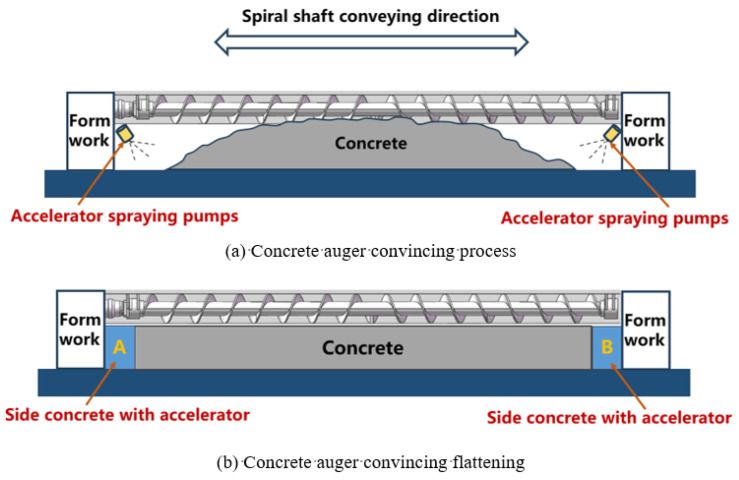
Slipform paver adds accelerator and flattens concrete working process.

To sum up, based on a slip form paver for roadway floor concrete (Figure 1), this paper realizes the rapid solidification forming of side concrete by adding the alkali-free accelerators to the concrete on both sides of the roadway floor to achieve the requirements of low collapse, no deformation, no displacement, and strength up to standard, thus improving the concrete formwork effect and floor forming quality. The specific sliding form paving process (Figure 2) is as follows: During the process of using the concrete sliding formwork paver shown in Figure 1 for spiral material distribution, the accelerator spraying pump is used to spray the accelerator on both sides of the concrete. After the concrete is evenly distributed and extruded out of the mold, the roadway floor structure is formed with two sides of accelerator-containing concrete (as shown in A and B in Figure 2), and the middle is ordinary concrete. This article aims to explore a new method of using accelerators to solve the problems of edge collapse, deformation, and displacement during concrete sliding mode paving.

In the aspect of concrete paving, many scholars have conducted a lot of research, focusing on concrete mix design, new concrete materials, new paving methods, and so on. Thomas Voigt and others optimized the concrete mixture ratio by changing the type and quantity of admixtures so that the concrete mixture consolidated without vibration and could maintain a certain shape in the sliding form pavement process [10]. J S Yeo and others used response surface methodology to optimize the concrete mix ratio of pavement paving and thought that the best water–binder ratio and bone–binder ratio were 0.35 and 3.50 [11]. Rakesh Kumar studied the effect of a recycled coarse aggregate of construction waste on the wear resistance of concrete with 0.38 and 0.44 water cement ratio [12]. Based on the unique advantages of magnesium phosphate cement and sodium bicarbonate as a foaming agent, Cong Ma and Bing Chen prepared a new type of foam concrete with the characteristics of the rapid setting and high early strength [13]. Ivana Barišíc and others compared the thermal properties of three different concrete paving materials [14]. Abbas Solouki and others used concrete containing waste silt for concrete pavements and carried out mix proportion optimization design [15]. Qiqi Tan and others systematically combed and prospected the frontier research of new prefabricated pavement technology [16].

There are many kinds of accelerators, which can be roughly divided into four categories: alkaline powder, alkaline-free powder, alkaline liquid, and alkaline-free liquid [17]. Among them, alkaline-free liquid accelerators are the most widely used due to their ease of addition, good initial setting effect, and minimal loss of strength in the later stage. Many scholars have performed a lot of research on the properties, preparation methods, and action mechanisms of accelerators. For example, Renhe Yang and others prepared the AFa accelerator and tested its performance, studied the effect of AFa on the early hydration of C3A and C3S, and established a hydration model [18]. Renhe Yang and Tingshu He studied the early hydration process of concrete under the combined action of different mineral admixtures (fly ash, S95 slag, and silica fume) and different liquid accelerators (AR, AS, and AF) and obtained the best dosage [19]. Yongdong Xu and others studied the influence of calcium sulphoaluminate cement on the hydration process of cement paste containing an alkali-free liquid accelerator, from the aspects of setting time, compressive strength, and hydration process, and obtained that the dosage range of calcium sulphoaluminate cement is 5–10% [20]. Yanping Sheng and others developed a new alkali-free liquid accelerator with good coagulation accelerating effect, and the best dosage was 8% [21]. Natallia Shanahan and others found that too high a dosage of the accelerator will reduce the compressive strength, tensile strength, and elastic modulus of concrete materials, and change the tensile and compressive ratio of materials [22]. J. Sathya Narayanan and others compared the effects of different types of accelerators in foam concrete and thought that C fly ash had the best effect, which could make concrete demold within 90 min [23]. Saikat Das and others prepared a new type of accelerator with agricultural waste as raw material, which can accelerate the hardening process of concrete and improve the early strength. Mixing into concrete can reduce the time of dismantling formwork and speed up the production cycle of concrete prefabricated parts [24]. Yifei Wang and others summarized the effects of different types of accelerators on the hydration, microstructure, and properties of cement-based materials and thought that most accelerators had negative effects on the durability of concrete when the dosage was too high [25].

At present, there is no related research on the application of accelerators in concrete paving of roadway floors. Sliding mode paving is more difficult compared to other paving methods that use accelerators because rail type, frame type, and other paving methods will lay a large number of templates to support the concrete, and the templates will be removed after the initial setting of the concrete, which requires lower performance indicators such as slump of the concrete. However, sliding formwork paving only has one sliding template and will not always support the concrete, so the sliding formwork paving method puts forward higher requirements for the performance indicators of concrete. Given the engineering practice, this paper selects the J85 and FSA-AF accelerators [26], through mechanical analysis and single factor experiment, from the initial setting time, slump, deformation resistance of concrete, and compressive strength—four aspects to determine the appropriate type of accelerator and the range of dosage—and then through orthogonal experiment to determine the best dosage of the accelerator and concrete water–cement ratio. Finally, this paper puts forward the estimation formula of side concrete width; determines the reasonable width of side concrete in engineering applications; and adopts the best accelerator dosage, water–cement ratio, and side width to carry out verification experiments. To sum up, this paper solves the problems of edge collapse, deformation, and displacement in concrete paving from two aspects of paving materials and dimensions.

## 2. Materials and Methods

### 2.1. Mechanical Analysis and Experimental Design

**Figure 3:**
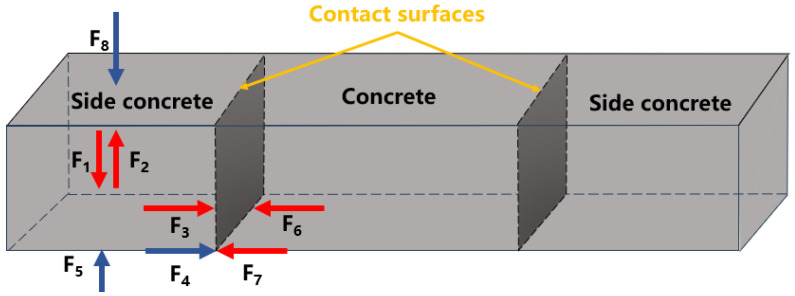
Force analysis of concrete with the accelerator on the side.

The reasonable dosage range of different types of accelerators is different. In order to facilitate the pumping of shotcrete, its water–cement ratio and slump are large, and the aggregate gradation is set for the spray flow. These characteristics are very different from the concrete used in road paving, and the performance index requirements of shotcrete and the index requirements of pavement concrete are quite different. Therefore, the dosage range of shotcrete cannot be directly applied to concrete paving [27]. By analyzing the acting force of concrete with the accelerator on the side in the paving process, the experimental items to be carried out can be made clear; then, the suitable range of accelerator dosage is determined, and the most suitable accelerator dosage and water–cement ratio for concrete paving are determined through orthogonal experiment [28]. Combined with the working characteristics of roadway floor concrete paving, a simple mechanical model of side concrete with accelerator is established, and its mechanical analysis is carried out in paving and working stages. In the analysis, the micro forces inside the concrete particles are not considered, only the macro forces on the surface of the concrete aggregates are considered. The stress analysis results of the side concrete are shown in Figure 3.

The blue arrow represents the external force acting on the side concrete, while the red arrow represents the internal force shown on the side concrete. F1 is the dead weight of concrete, F2 is the internal supporting force of concrete, F3 is the deformation resistance of concrete, F4 is the ground friction force, F5 is the ground supporting force, F6 is the local side pressure exerted by the middle concrete, F7 is the overall side pressure exerted by the middle concrete, and F8 is the vehicle pressure (F8 = 0 in paving stage).

Combined with the actual working conditions, the stress analysis includes the following:

Concrete paving stage: F1 = F2 + F5, F3 = F6, F4 = F7;

Working stage: F1 + F8 = F5.

Combined with the results of force analysis, the corresponding experimental items and sequences are determined according to different forces to carry out the experiments.

1. F1, concrete dead weight—initial setting time and slump experiment: Need shorter initial setting time and smaller slump. Because F1 corresponds to the ability of concrete to maintain its shape in a short period of time, only the time required for concrete to lose its plasticity and have a certain strength (initial setting time) is assessed, and the final setting time experiment is not considered.
2. F3, the concrete deformation resistance, and F6, the local side pressure exerted by the middle concrete—deformation resistance and side pressure experiment: With pressure (KN/m^2^) as the testing unit, the pressure change in the process of 0–2 mm compression deformation of concrete after the molding (the maximum pressure is taken as the deformation resistance of the concrete) and the pressure of the middle concrete of the side concrete at different heights are measured to determine the dosage range of accelerating agent that meets the deformation requirements.
3. F4, friction force, and F7, integral side pressure—concrete displacement problem: Ensure F4 friction force ≥ F7 integral side pressure, and avoid the problem of sliding displacement of concrete on both sides after being pressed by middle concrete. Put forward the width estimation formula to determine the appropriate side concrete width.
4. F8 vehicle pressure—28 d compressive strength test: Test the long-term performance of concrete after adding accelerator, and ensure that the 28 d compressive strength of concrete containing accelerator meets GB/T 28635-2012 “Precast concrete paving units” [29].

To sum up, this paper needs to carry out the above four aspects of experiments to ensure that the dosage range of the accelerator meets the requirements of rapid solidification, low collapse, no deformation, no displacement, and strength of side concrete.

**Figure 4:**
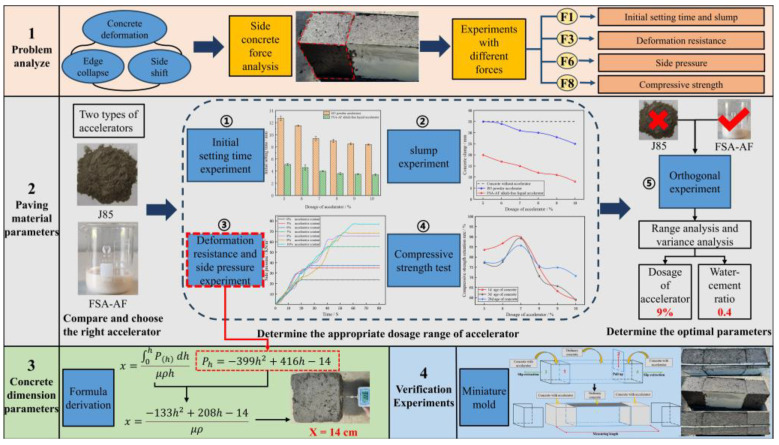
Experimental flow chart.

According to the results of the mechanical analysis, the overall experimental flow chart is designed as follows in Figure 4.

### 2.2. Experimental Materials

The cement is PO42.5 ordinary Portland cement; the particle size of sand is 0.5–2.0 mm; the particle size of the stone is 5mm; and the mixture ratio of cement, sand, and stone is 1:1.5:2.25. Cement, stones, sand, vicat instrument and slump bucket are all purchased in the Chinese market (Qingdao, Shandong, China). The dosage of the accelerator is 5–10%; the water–cement ratio is 0.4–0.5; the water–cement ratio is 0.45 in single factor experiment; and the water–cement ratio is 0.4, 0.45, and 0.5 in the orthogonal experiment [30,31,32].

**Figure 5:**
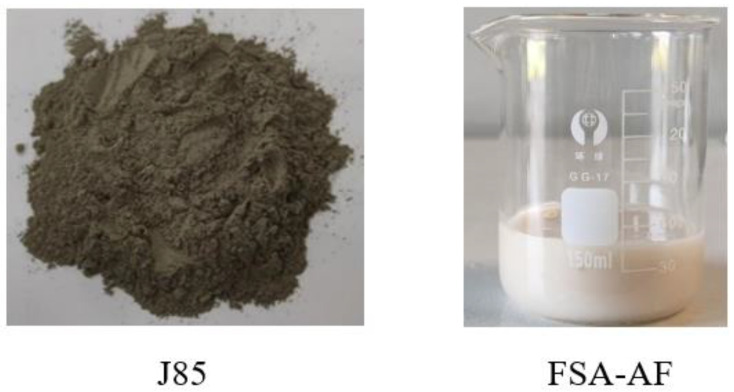
Samples of two different types of accelerators.

Inorganic salt powder accelerator has less dosage, high economy, and easy addition; alkali-free liquid accelerator has large dosage, high price, and better accelerator effect, but it is necessary to pay attention to the influence of its water dosage on the water–cement ratio of concrete [19]. Therefore, according to the different characteristics of the two accelerators, the J85 powder accelerator and FSA-AF alkali-free liquid accelerator are selected (as shown in Figure 5), and the performance of the two accelerators in concrete paving materials is compared experimentally. Both of the accelerators are commonly used in coal mine underground engineering and can be purchased in the Chinese market (Qingdao, Shandong, China). The main components of J85 powder accelerator are inorganic salts such as sodium aluminate, and the main components of FSA-AF alkali-free liquid accelerator are aluminum sulfate, hydrofluoric acid, and aluminum hydroxide. Their performance meets the requirements of GB/T35159-2017 “Flash setting admixtures for shotcrete” [33].

### 2.3. Initial Setting Time and Slump Experiment

In the initial setting time experiment, the Vicat instrument was used to measure the setting time of cement paste, and the test was carried out according to the requirements of GB/T 35159-2017 “Flash setting admixtures for shotcrete” [33]. The specific dosage of each raw material is shown in Table 1:

In the slump experiment, the standard slump bucket was used to test, and the mixture ratio of cement, sand, and stone was 1:1.5:2.25. The specific dosage of each raw material is shown in Table 2:

### 2.4. Deformation Resistance and Side Pressure Experiment of Concrete Containing Accelerator

The pressure on concrete is an important reason that affects the deformation and displacement of side concrete. Previous studies have conducted a lot of research on this, including the influence of temperature, slump, setting time on concrete pressure, variation characteristics of concrete on the side pressure of large-scale members, pressure prediction models, etc. However, there are few studies on the deformation resistance of concrete in the paving process (height 0~40 cm), and there is no ready-made instrument to use [34,35,36,37].

**Figure 6:**
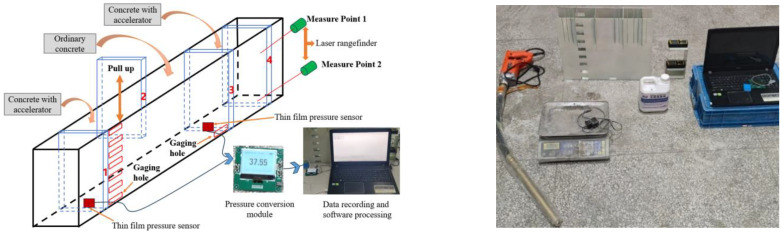
Experimental devices for deformation resistance and side pressure of concrete.

Therefore, combined with the characteristics of concrete paving, an experimental device is designed, which can measure the deformation resistance of concrete and the side pressure of concrete at different heights. In order to facilitate the experiment and data measurement, the experimental device has been reduced in size according to the characteristics of roadway floor paving. The device is made of steel plate with a height of 40 cm, a width of 10 cm, and a length of 50 cm. The left side of the device is provided with 8 evenly distributed measuring holes with a spacing of 3 cm to facilitate side pressure measurement. The device is provided with 4 withdrawable templates, and grooves are provided on the device to facilitate the change of template position for different measurements. Pressure measurement adopts MY2801 thin film pressure conversion template, which can convert the pressure of thin film into electrical signal output. The thin film pressure sensor is a circular gasket with a radius of 1.5 cm. MY2801-SSCOM-V5.13.1 software is used to collect and record data. The instruments and materials used in the experiment are all manufactured or purchased by Yanbotaike Technology Corporation (Qingdao, Shandong, China). The laser rangefinder is DL4168 with a detection accuracy of 1mm. Both pressure-measuring devices and laser rangefinders are available in the Chinese market and have been calibrated for accuracy, as shown in Figure 6.

**Figure 7:**
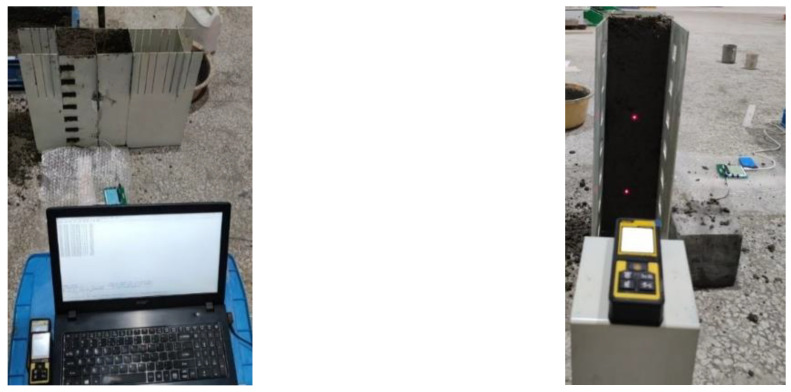
Experimental process of deformation resistance and side pressure of concrete.

Deformation resistance experimental flow (Figure 7): Concrete-containing accelerator is filled between No. 3 and No. 4 formwork, and No. 3 and No. 4 formwork is extracted after initial setting. The membrane pressure sensor is placed on the inner side of the concrete, and two laser rangefinders are placed on the upper and lower sides of the outer side. Then, add ordinary concrete evenly multiple times to the area between the No. 2 formwork and the concrete column, and lightly compact it with a vibrator. When the difference of laser measuring points is 2 mm, stop adding concrete, record the process pressure value, and take the value when the deformation is 2 mm as the deformation resistance value. The deformation amount is according to the relevant requirements in GB/T 29013-2012 “Road construction and road maintenance machinery and equipment—Slipform paver” [38] and GB/T 28635-2012 “Precast concrete paving units” [29].

Experimental flow of concrete side pressure (Figure 7): Put the membrane pressure sensor into the measuring holes with different heights on the left side of the device, fill the ordinary concrete square column with a height of 40 cm between the No. 1 and No. 2 formwork, vibrate and compact with a vibrating rod, and record the side pressure values under the measuring holes with different heights.

The deformation resistance and lateral pressure of concrete are characterized by the pressure value (KN/m^2^).

### 2.5. Compressive Strength Experiment of Concrete

While accelerating the setting of concrete, the accelerator will have a certain impact on the later strength of the concrete. Excessive dosage will lead to a large loss of strength and even shrinkage and cracking, which will affect the service life of concrete [22,25].

Therefore, the compressive strength of concrete at five ages of 1 d, 3 d, 7 d, 14 d, and 28 d with different dosages is tested by a universal pressure tester (Qingdao, Shandong, China), and the variation law between the dosage of the accelerator and the compressive strength of concrete is explored. Combined with the previous experimental results, the appropriate dosage range of the accelerator is finally determined.

### 2.6. Orthogonal Experiment

The content of accelerator and water–cement ratio are two important factors that affect the quality of concrete sliding form paving. In order to ensure the best effect of paving concrete, it is necessary to determine the best parameters within the corresponding range of the two factors, and the orthogonal experiment method can screen out the best multi-factor and multi-level parameter combination. Therefore, taking the dosage of accelerator and water–cement ratio as factors, the orthogonal experiment of two factors and three levels is carried out within the range of the dosage and water–cement ratio of accelerator determined by pre-experiment, and the performance indexes of concrete under different combinations are determined. According to the experimental results, the optimal dosage and water–cement ratio of the accelerator are determined.

### 2.7. Estimation Formula of Side Concrete Width

Because the concrete with an accelerator on the side can set quickly and has a certain strength, the ordinary concrete without setting in the middle will exert pressure on the side concrete, which may lead to the displacement of the concrete on both sides. Based on mechanical analysis and calculus, the formula for estimating the width of side concrete is put forward, which can be used to estimate the width of side concrete that meets the requirements.

### 2.8. Validation Experiment

**Figure 8:**
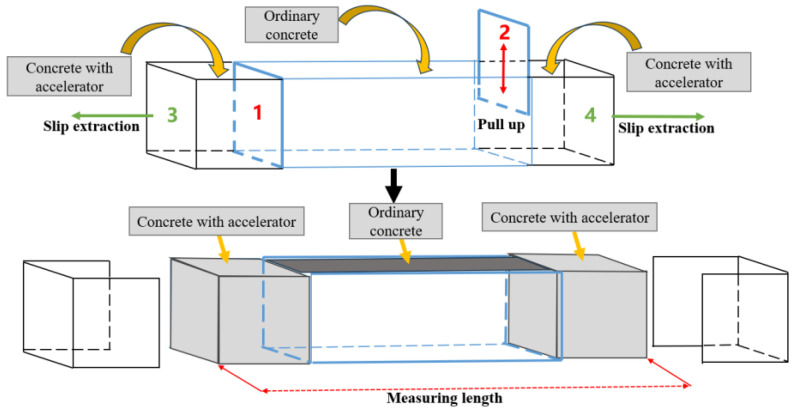
Schematic diagram of verification experimental mold device.

The overall width of the experimental device is 15 cm, with a maximum paving height of 40 cm. The length of the molds on both sides is 14 cm, and the length of the middle mold is 32 cm. The middle template (indicated by numbers 1 and 2) can be pulled out up and down, and the molds on both sides (indicated by numbers 3 and 4) can be slid out to both sides. Using a small mold (Figure 8) to carry out the verification experiment, according to the dosage of accelerator of 9% and water–cement ratio of 0.4 to prepare concrete containing accelerator, fill in the side mold; fill in the middle part of ordinary concrete; draw out No. 1 and No. 2 formworks; vibrate and compact with a vibrator; remove No. 3 and No. 4 formworks; compare the length before and after demolding; observe the shape of side concrete; and verify whether there are problems of edge collapse, deformation, and displacement. The instruments and materials used in the experiment are all manufactured or purchased by Yanbotaike Technology Corporation (Qingdao, Shandong, China).

## 3. Results

### 3.1. Initial Setting Time and Slump

**Figure 9:**
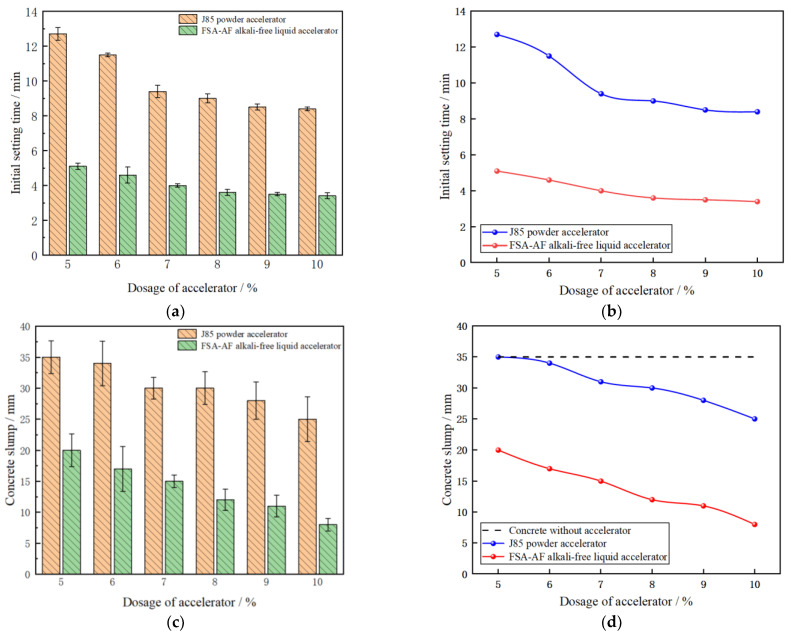
(**a**) Comparison chart of initial setting time. (**b**) Change trend chart of initial setting time. (**c**) Slump comparison chart. (**d**) Slump change trend chart.

The initial setting time of cement paste and concrete slump after adding the J85 powder accelerator and FSA-AF alkali-free liquid accelerator is shown in Figure 9.

Figure 9a shows that the initial setting time of the two accelerators is shortened with the increase in the dosage. The initial setting time of J85 powder accelerator is 12.7 min at 5% dosage, 8.4 min at 10% dosage, 5.1 min at 5% dosage, and 3.4 min at 10% dosage of FAS-AF alkali-free liquid accelerator. Therefore, the accelerating effect of the FSA-AF alkali-free liquid accelerator is far better than that of the J85 powder accelerator. Yongdong Xu came to a similar conclusion when studying the effects of inorganic salt accelerators on the properties of different types of concrete. It is speculated that the reason is that the inorganic salt component contained in the J85 powder accelerator and PO42.5 ordinary Portland cement have poor adaptability and a weak promotion effect on cement hydration [39,40].

Figure 9b shows that after the dosage of two accelerators reaches 8%, the trend of initial setting time in the J85 accelerator was 9 min, 8.5 min, and 8.4 min, and the trend of initial setting time in FAS-AF accelerator was 3.6 min, 3.5 min, and 3.4 min. This shows that the changing trend of initial setting time tends to be flat with the increase in dosage, indicating that after the dosage exceeds 8%, the improvement effect of accelerators on initial setting time weakens [41].

Figure 9c,d show that the slump of concrete decreases with the increase in accelerator dosage: the slump of ordinary concrete is 35 mm, the slump of J85 accelerator is 35 mm at 5% and 25 mm at 10%, and the slump of FSA-AF accelerator is 20 mm at 5% and 8 mm at 10%. So, the slump of concrete containing FSA-AF alkali-free liquid accelerator is lower than that of concrete containing J85 powder accelerator.

To sum up, the initial setting time and slump data of the FSA-AF alkali-free liquid accelerator are far better than those of the J85 powder accelerator in paving. Sliding mode paving requires concrete to have the characteristics of rapid solidification and low slump; so, the J85 powder accelerator was eliminated and FSA-AF alkali-free liquid accelerator was selected. Therefore, further experiments will not be conducted on the J85 powder accelerator, and the “accelerator” mentions in the following text all represent the FSA-AF alkali-free liquid accelerator. It can be seen from Figure 9b,d that the variation trend of the initial setting time of the FAS-AF accelerator tends to be flat within the range of 8–10% and reaches the lowest level. The collapse value of FSA-AF accelerator is significantly lower than that of the J85 accelerator in the range of 8–10%, all at a low level. Therefore, according to the trend of initial setting time and slump curve, considering the short initial setting time and low slump of FAS-AF type alkali-free liquid accelerator, the dosage of FSA-AF alkali-free liquid accelerator should be 8~10%.

### 3.2. Deformation Resistance and Side Pressure

**Figure 10:**
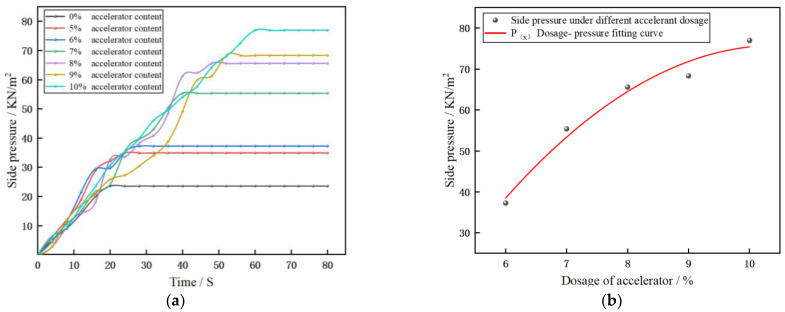

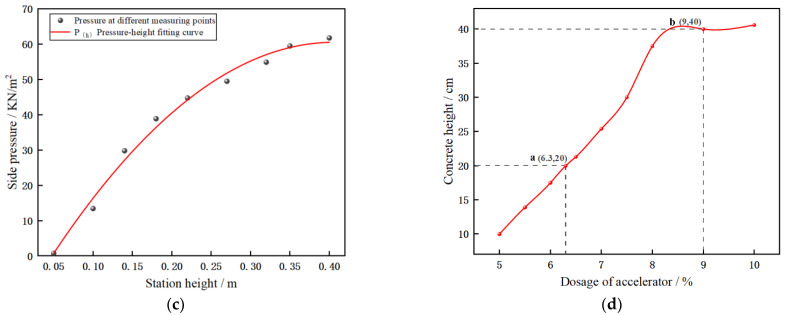
(**a**) Pressure change during concrete deformation. (**b**) Fitting curve of dosage–deformation resistance. (**c**) Side pressure–height fitting curve. (**d**) Dosage–height curve.

Figure 10a shows that the deformation resistance of concrete is positively correlated with the dosage of the FSA-AF alkali-free liquid accelerator. Compared with ordinary concrete, the deformation resistance of concrete after adding the FSA-AF accelerant is significantly improved. Take the peak pressure in Figure 10a as the discrete point, where the discrete point corresponding to the 5% coagulant dosage has a significant difference from other points. The pressure corresponding to the 5% dosage point is smaller than that in the 7~10% dosage range, and there is no obvious difference from the neighboring 6% dosage point, which cannot reflect the change trend of the overall fitting curve well. If the 5% dosage point is added to the fitting curve, it may lead to a large error in the fitting curve, which will affect the following results. So, to ensure the excellent fitting effect of the curve, the discrete points corresponding to the 5% dosage were discarded, and a fitting curve (Figure 10b) was plotted between the concrete deformation resistance and the dosage of the FSA-AF accelerator.

Figure 10b shows that the polynomial fitting relationship between the deformation resistance of concrete (*P*) and the dosage of the FSA-AF accelerator (*x*) is as follows:

(1) $$P_{x}=-1.9x^{2}+39x-130$$

The value of R^2^ is 0.98 > 0.9, which indicates that there is an excellent correlation between the experimental data and the fitting curve.

The error range of fitting relation is

(2) $$P_{x}=-1.9{(\pm 0.9)x}^{2}+39(\pm 14)x\;-\;130(\pm 55)$$

Figure 10c shows that the side pressure of concrete is positively correlated with the paving height of concrete, and the polynomial fitting relationship between the side pressure and the paving height is as follows:

(3) $$P_{h}=-471h^{2}+383h-\;17$$

The R^2^ value is 0.99 > 0.9, indicating that there is an excellent correlation between the experimental data and the fitting curve [42,43].

The error range of fitting relation is

(4) $$P_{h}=-471(\pm 72)h^{2}+383(\pm 33)h\;-\;17(\pm 3)$$

From the error range of Formulas (2) and (4), it can be seen that in the commonly used 20~40 cm concrete paving range, the fluctuation in the error range has little influence on the results of the fitting curve. Therefore, considering the engineering redundancy, a larger value can be taken within the error range.

According to the fitting curves (1) and (2) obtained in Figure 10b,c, the *h x* (height–accelerator dosage) curve (Figure 10d) for the same deformation resistance and side pressure can be drawn. According to the curve in Figure 10d, it can be determined that the appropriate dosage range of the FSA-AF accelerator within the height of 20~40 cm (commonly used height range for floor paving) is 6.3~9% (As shown by point a and b in Figure 10d).

### 3.3. Compressive Strength

**Figure 11:**
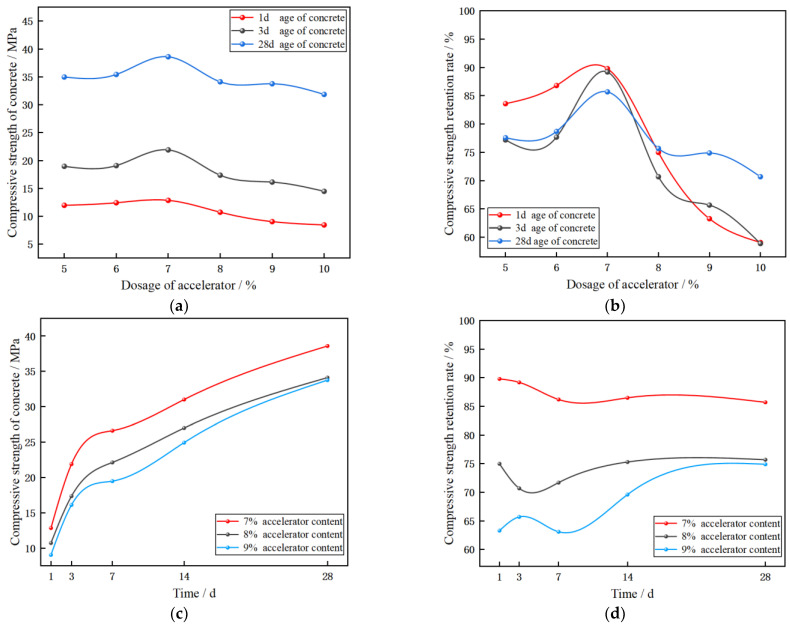
(**a**) Curve of compressive strength with dosage. (**b**) Curve of strength retention rate with dosage. (**c**) Curve of compressive strength with time. (**d**) Curve of strength retention rate with time.

From Figure 11a,b, it can be seen that the compressive strength and retention rate of concrete with different accelerator dosages (the ratio of compressive strength of concrete containing accelerator to compressive strength of ordinary concrete) have the same change trend at 1, 3, and 28 days old, and they all increase at first and finally decrease with the increase in accelerator dosage, and the compressive strength and retention rate of concrete with 7% dosage are the highest. This shows that too high a dosage of the accelerator will lead to the loss of concrete strength, and the loss of concrete strength is the largest when the dosage of the accelerator is 10%, which is similar to the previous research law [44].

From Figure 11c,d, it can be seen that in the range of 7~9%, the 28-day compressive strength of concrete is over 70% and the compressive strength increases rapidly with the increase in age. The 28-day compressive strength meets the requirement that the compressive strength is not less than 25 Mpa specified in GB/T 28635-2012 “Precast concrete paving units” [29]. Therefore, considering the compressive strength, the optimal dosage range should be 7~9%.

The initial setting time and slump experiments determine the dosage range of 7~10%, deformation resistance and side pressure experiments determine the dosage range of 6.3~9%, and compressive strength experiments determine the dosage range of 7~9%.

Combined with the above experimental conclusions, it is confirmed that the reasonable range of accelerator is 7~9%.

### 3.4. Orthogonal Experimental Results

In the range of 7~9% accelerator dosage and 0.4~0.5 water–cement ratio, the performance indexes of concrete under different combinations are determined by orthogonal experiment, and the optimal combination is determined. The factor level of water–cement ratio is 0.4, 0.45, and 0.5; the factor level of accelerator content is 7%, 8%, 9%; and all of these are expressed by the numbers 1, 2, and 3. The results of the orthogonal experiment are shown in Table 3.

**Figure 12:**
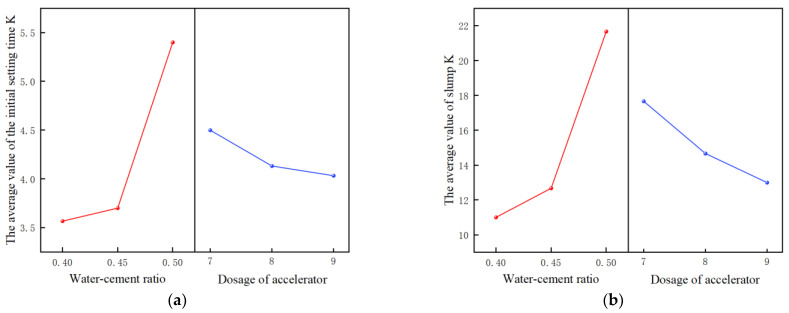

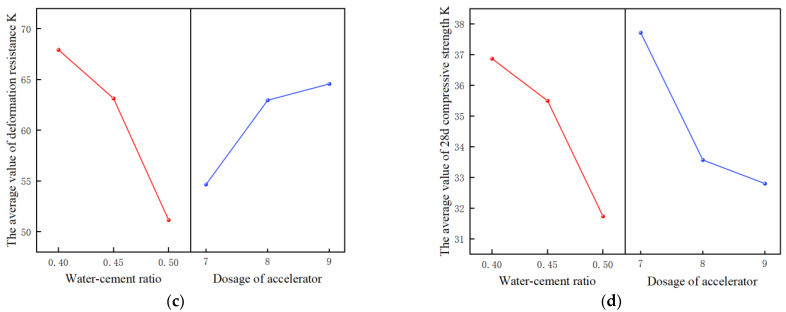
(**a**) Orthogonal effect curve of initial setting time. (**b**) Orthogonal effect curve of slump. (**c**) Orthogonal effect curve of deformation resistance. (**d**) Orthogonal effect curve of compressive strength.

The range analysis of orthogonal experimental results is carried out (Table 4), and the analysis results are drawn as orthogonal effect graphs (Figure 12).

Combined with Figure 12, the optimal combination of each index is selected: if the index requirement is small, the parameter combination with the minimum K value is selected, and on the contrary, the parameter combination with the maximum K value is selected. Through range analysis, Table 5 shows the influence degree of water–cement ratio and accelerator yield on each evaluation index and obtains the optimal level combination corresponding to each evaluation index.

Multivariate analysis of variance is used for the significance test to judge the significance of factors to evaluation indexes, to determine the optimal parameter combination of overall indexes. Based on the results of the range analysis, a variance analysis was conducted to determine the parameter combination that can achieve the optimal level of the four evaluation indicators. The variance analysis results are shown in Table 6. ** indicates factors with significant influence, *** indicates factors with extremely significant influence, and “—" indicates factors with insignificant influence.

The water–cement ratio has a particularly significant impact on the four evaluation indexes [45], and they all think that a 0.4 water–cement ratio is an excellent level; so, 0.4 is selected. The effect of the dosage of the accelerator on the deformation resistance and 28-day compressive strength is particularly significant, but the R-value of the deformation resistance is greater, so the dosage of the accelerator is 9%. It can be seen from the *p* value in Table 6 that the influence degree of the water–cement ratio on the four evaluation indicators is greater than that of the accelerator content, because the addition of the accelerator only speeds up the condensation of material particles in the concrete itself, without introducing new moisture or materials. The water–cement ratio is a key factor affecting the performance of concrete, and the difference in water content will seriously affect the strength and slump of concrete. Therefore, compared with the water–cement ratio, the influence of accelerator content is weak.

To sum up, the optimal parameter combination of the orthogonal experiment is as follows: water cement ratio 0.4; accelerator dosage 9%. In practical engineering application, the optimum combination of 0.4 water–cement ratio and 9% accelerator dosage is adopted, and the concrete has a fast initial setting time and good late strength. As a result, the concrete can slide out of the mold faster, which increases the paver’s travel speed and work efficiency. At the same time, good post-strength can be more convenient for the daily use and maintenance of roadway floor, reducing the cost of use.

### 3.5. Derivation of Formula for Estimating the Width of Side Concrete

**Figure 13:**
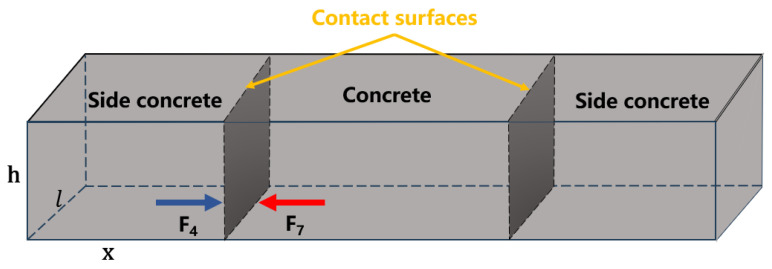
Simple model of side force.

Set the side concrete length l, height h (0.2–0.4 m), width x, side concrete quality m, density ρ, and ground friction coefficient μ. The force analysis results are shown in Figure 13.

Without considering micro forces such as cohesion between concrete particles. In equilibrium, we have the following:

The friction force *F*_4_ at the bottom of the side concrete is equal to the pressure *F*_7_ exerted by the middle concrete.

(5) $$F_{4}={\;F}_{7}$$

F_4_: Use the formula

(6) $$F_{4}=\mu mg=\mu \rho vg=\mu \rho lxhg$$

F_7_: Use the formula

(7) $$F_{7}=PS=P\;\times \;l\;\times \;h$$

Integral calculation of the overall side pressure of concrete includes

(8) $$F_{7}=\int_{0}^{h}P_{h}l\;dh\;=F_{4}=\;\mu \rho lxh$$

Simplification includes

(9) $$x\;=\frac{\int_{0}^{h}P_{h}\;dh}{\mu \rho hg}$$

In the formula, ρ is the density of side concrete kg/m^3^, l is the length of side concrete m, x is the width of side concrete m, h is the height of side concrete m, μ is the friction coefficient between the side concrete and ground, and $P_{h}$ is the relationship between side pressure and concrete height.

The relation $P_{h}$ has been obtained through the pressure–height relation experiment. To reserve redundancy in engineering applications, the maximum pressure value is taken within the error range, and the corresponding relation $P_{h}$ is

(10) $$P_{h}=-399h^{2}+416h-14$$

Incorporated into Formula (8), the estimation formula of side concrete width can be obtained by simplification,

(11) $$x=\frac{-133h^{2}+208h-\;14}{\mu \rho g}$$

According to the formula, it can be seen that the width of the side concrete is the key to ensuring the smooth paving of concrete slip form, and the length, material, and section size ratio of formwork have no influence [46,47].

According to the actual engineering situation, the ground friction coefficient is 0.02, the concrete density is 1800 kg/m^3^, the maximum paving height is 0.4 m, and the required width of side concrete can be obtained by bringing it into Formula (10):

X ≈ 0.133 m ≈ 14 cm

### 3.6. Verification Experiments

The designed experimental device is used to verify the optimal parameter combination and the width of side concrete.

Firstly, the performance index of concrete is tested to verify the correctness and practicability of the results obtained by orthogonal experiment. Concrete is prepared by the best combination of parameters, and various performance indexes are tested to verify the orthogonal experimental results (Table 7).

Under the optimal combination of parameters, the initial setting time and slump values are smaller, and the deformation resistance and 28-day compressive strength values are larger. Compared with the previous experimental results, it can be seen that they are at the optimal level.

**Figure 14:**
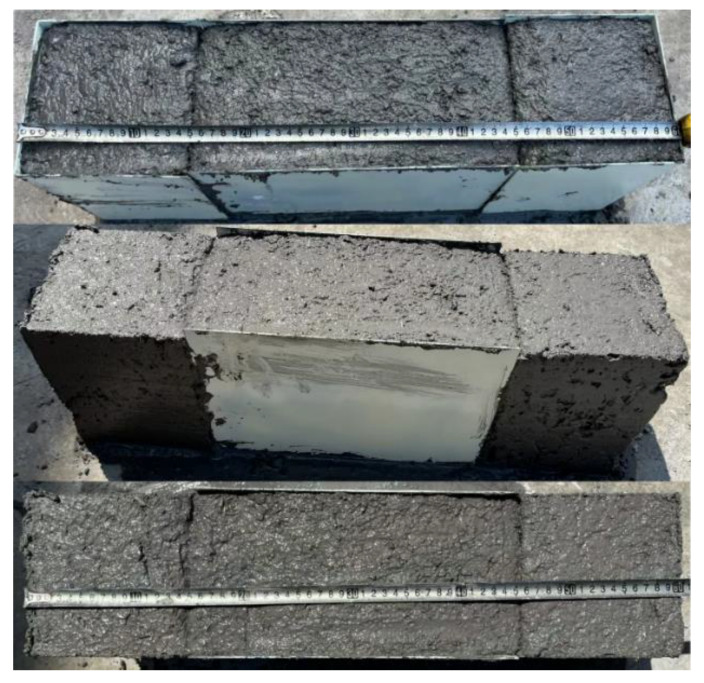
Verification experiment process.

After the orthogonal experiment results are verified, the problems of side concrete collapse, deformation, and displacement are verified. As shown in Figure 14, after removing the molds on both sides, the side concrete has good shape and surface flatness, and there is no edge collapse and deformation problem. The length before and after formwork removal is the same as that measured by tape measure, which is 60 cm, indicating that there is no displacement problem of side concrete.

Therefore, the optimum parameters obtained in this paper, such as the dosage of the accelerator 9%, water–cement ratio 0.4, and the width of side concrete 14 cm, can be applied to practice and achieve good results.

## 4. Conclusions

To solve the problems such as edge collapse, deformation, and displacement in roadway floor paving, through mechanical analysis and single factor experiments, this paper determines the suitable type and dosage range of the accelerator from four aspects: initial setting time, slump, deformation resistance of concrete, and compressive strength; then, the best dosage of the accelerator and the water–cement ratio of concrete is determined through orthogonal experiment. In this paper, a formula for estimating the width of side concrete is proposed, and the reasonable width of side concrete is obtained.

The main conclusions are as follows:

(1) FSA-AF alkali-free liquid accelerator can meet the performance requirements of roadway floor paving. The initial setting time and slump data of the FSA-AF alkali-free liquid accelerator are far better than those of the J85 powder accelerator. Compared to other accelerators, the FSA-FA accelerator has a shorter initial setting time, lower slump, and higher deformation resistance and compressive strength. In concrete sliding mode paving, the FSA-FA accelerator can better ensure that the side concrete does not collapse or deform.
(2) The suitable range is the water–cement ratio of 0.4~0.5 and the dosage of the FAS-AF accelerator is 7~9%. The water–cement ratio of 0.4 and the FAS-AF accelerator dosage of 9% are the best parameter combination.
(3) The relationship between the deformation resistance of concrete and the dosage of the FSA-AF accelerator is as follows:

$$P_{x}=-1.9x^{2}+39x-130$$

(4) The relationship between concrete side pressure and paving height is as follows:

$$P_{h}=-471h^{2}+383h-17$$

(5) According to the estimation formula of side concrete width, the side width should be 14 cm, and the estimation formula of side concrete width is

$$x=\frac{-133h^{2}+208h\;-\;14}{\mu \rho g}$$

(6) Adding accelerant to the concrete on both sides can solve the problems of collapsing edge, deformation, and displacement of concrete sliding form paving, and good results were obtained through verification experiments. The side concrete has a good ability to ensure its own shape and surface smoothness, and the performance index of concrete reached the expected effect.

The key parameters of paving materials and dimensions summarized by the above conclusions in this paper can provide a parameter basis and theoretical reference for the concrete paving of the roadway floor in an underground coal mine and can effectively solve the problems of side concrete collapse, deformation, and displacement existing in underground concrete paving at present, ensuring the rapid formation of concrete and the ability to maintain shape. The paving quality and efficiency of the roadway floor are improved, paving cost and paving difficulty are reduced, and the research has good engineering application value.

## Figures and Tables

**Table 1:** Dosage of each material in the initial setting time experiment.

Accelerator Dosage	Weight (g)			
	Water J85/FSA-AF	Cement	J85 Powder Accelerator	FSA-AF Alkali-Free Liquid Accelerator
5%	225/212.5	500	25	25
6%	225/210.0		30	30
7%	225/207.5		35	35
8%	225/205.0		40	40
9%	225/202.5		45	45
10%	225/200.0		50	50

**Table 2:** Dosage of each material in slump experiment.

Accelerator Dosage	Weight (g)					
	Water J85/FSA-AF	Cement	Sand	Stones	J85 Powder Accelerator	FSA-AF Alkali-Free Liquid Accelerator
5%	1350/1275	3000	4500	6750	150	150
6%	1350/1260				180	180
7%	1350/1245				210	210
8%	1350/1230				240	240
9%	1350/1215				270	270
10%	1350/1200				300	300

**Table 3:** Orthogonal experimental results.

Serial Number	Factor A Water–Cement Ratio	Factor B Dosage of Accelerator	Initial Setting Time (min)	Slump (mm)	Resistance to Deformation (KN/m^2^)	28 d Compressive Strength (MPa)
1	0.40	7	3.7	13	62.95	39.80
2	0.40	8	3.5	10	69.92	35.55
3	0.40	9	3.5	10	70.89	35.26
4	0.45	7	4.0	15	55.40	38.61
5	0.45	8	3.6	12	65.63	34.13
6	0.45	9	3.5	11	68.35	33.77
7	0.50	7	5.8	25	45.64	34.76
8	0.50	8	5.3	22	53.32	31.04
9	0.50	9	5.1	18	54.48	29.38

**Table 4:** Range analysis.

Evaluation Indicators	Factor A Water–Cement Ratio			Factor B Dosage of Accelerator			R	
	$\bar{K}$1	$\bar{K}$2	$\bar{K}$3	$\bar{K}$1	$\bar{K}$2	$\bar{K}$3	RA	RB
Initial setting time	3.567	3.700	5.400	4.500	4.133	4.033	1.833	0.467
Slump	11.000	12.670	21.670	17.670	14.670	13.000	10.670	4.670
Resistance to deformation	67.920	63.130	51.150	54.660	62.960	64.570	16.770	9.910
28 d compressive strength	36.870	35.500	31.730	37.720	33.570	32.800	5.140	4.920

**Table 5:** Optimal combination of each index.

Evaluation Indicators	Engineering Requirements	Factor Primary	Excellent Level	Factor A Water–Cement Ratio	Factor B Dosage of Accelerator
Initial setting time	The smaller	A, B	A1B3	0.4	9%
Slump	The smaller	A, B	A1B3	0.4	9%
Resistance to deformation	The larger	A, B	A1B3	0.4	9%
28 d compressive strength	The larger	A, B	A1B1	0.4	7%

**Table 6:** Analysis of variance.

Response Factors	Factors	Class III. Sum of Squares	Degree of Freedom	Mean Square Deviation	F	*p*	Distinctiveness
Initial setting time	Water–cement ratio	6.269	2	3.134	194.552	<0.001	***
	Accelerator dosage	0.362	2	0.181	11.241	0.023	**
Slump	Water–cement ratio	197.556	2	98.778	68.385	0.001	***
	Accelerator dosage	33.556	2	16.778	11.615	0.22	—
Resistance to deformation	Water–cement ratio	447.841	2	223.921	122.197	<0.001	***
	Accelerator dosage	169.601	2	84.801	46.277	0.002	***
28 d compressive strength	Water–cement ratio	42.603	2	21.301	138.254	<0.001	***
	Accelerator dosage	42.029	2	21.014	136.391	<0.001	***

**Table 7:** Verification results of orthogonal experiment.

Optimal Combination	Initial Setting Time (min)	Slump (mm)	Resistance to Deformation (KN/m^2^)	28d Compressive Strength (MPa)
(0.4, 9%)	3.600	8.000	73.027	34.669

## Data Availability

The original contributions presented in this study are included in the article material. Further inquiries can be directed to the corresponding author.
